# Optical phased arrays for wavefront shaping in forward scattering media

**DOI:** 10.1515/nanoph-2025-0273

**Published:** 2025-09-22

**Authors:** Filip Milojković, Niels Verellen, Roelof Jansen, Frédéric Peyskens, Xavier Rottenberg, Pol Van Dorpe

**Affiliations:** IMEC, Kapeldreef 75, 3001 Leuven, Belgium; Department of Physics and Astronomy, KU Leuven, 3001 Leuven, Belgium

**Keywords:** integrated photonics, wavefront shaping, scattering media, optical phased array, silicon photonics, biomedical imaging

## Abstract

High-resolution optical imaging in thick tissue samples remains elusive, mainly because of the scattering exhibited by the tissue. With increasing depth, the number of nonscattered photons exponentially decreases – limiting the use of conventional imaging techniques at depth. Wavefront shaping is a novel technique that aims to enable imaging at depth by refocusing the scattered light. However, significant wavefront-control hardware improvements are necessary to unlock the applications in *in vivo* microscopy. Optical phased arrays (OPAs), realized in integrated photonics, can provide improvements in the pixel pitch, operation speed, and system compactness compared to conventionally employed spatial light modulators. We compare different OPA designs for focusing in tissue-like forward-scattering samples. OPA design trade-offs, such as the array pitch, number of antennas, and antenna emission profile, are experimentally studied, and their influence on the device performance is highlighted. We do this for increasing thickness of the forward-scattering sample and observe two distinct regimes. The devices, operating at the wavelength of *λ* = 852 nm, were fabricated on a SiN photonics platform suitable for both near-infrared (NIR) and visible (VIS) light.

## 1 Introduction

**Figure 1:**
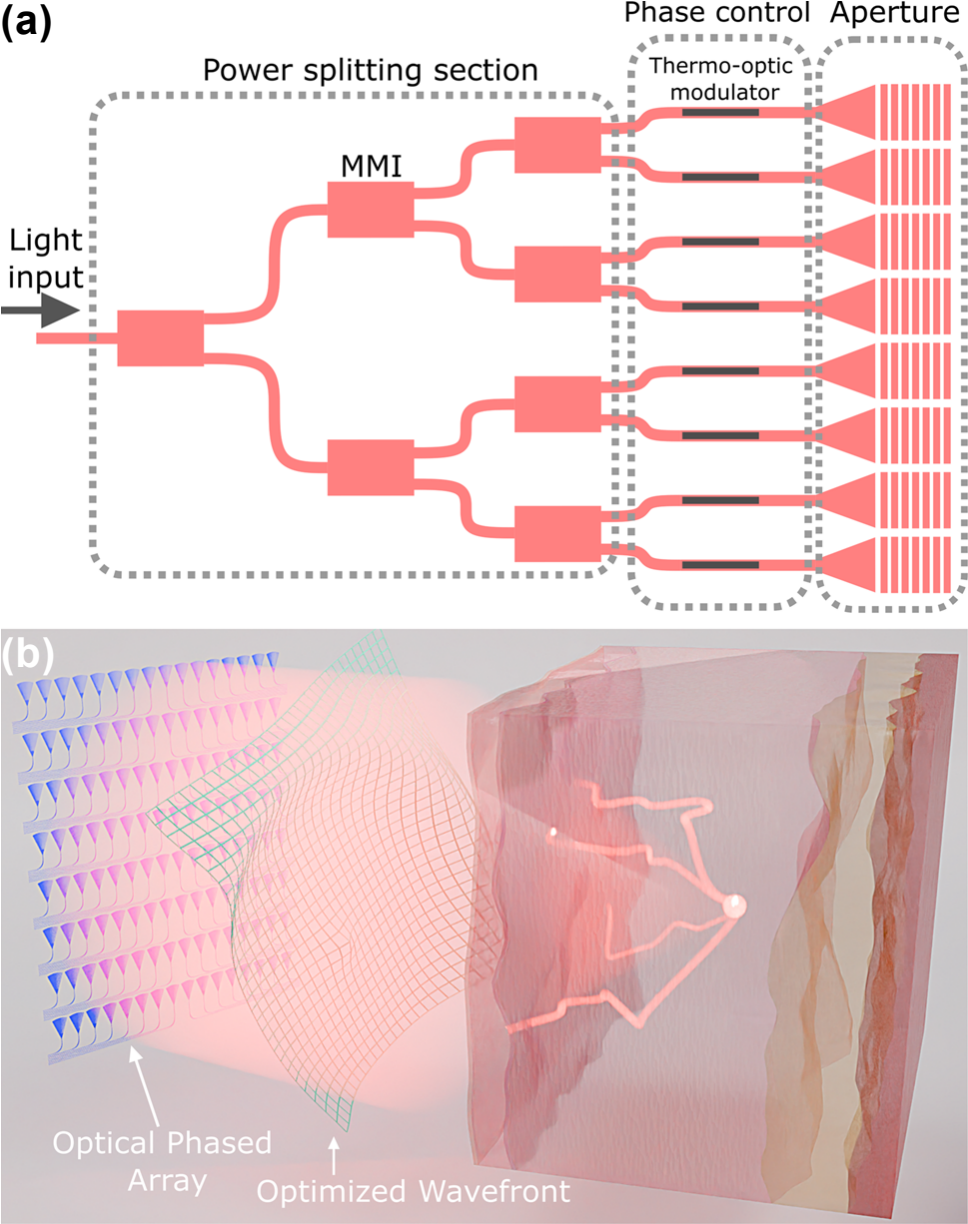
Wavefront shaping device and principle. (a) Illustration of the OPA building blocks. Power splitting section relies on multimode interferometers (MMIs), while the phase control is achieved using thermo-optic modulators with characterized efficiency of 20 mW/*π*. OPA aperture consists of diffraction gratings, which radiate the light out of the PIC. (b) Wavefront shaping principle illustration. OPA emits an optimized illumination wavefront, resulting in the focusing of scattered photons.

The maximum imaging depth of optical microscopy techniques is limited by the light scattering exhibited by the tissue. While the majority of imaging techniques tends to suppress the scattered photons, wavefront shaping aims to enable imaging at greater penetration depths by controlling the scattered light. By optimizing the illumination wavefront using a spatial light modulator (SLM), i.e., by performing the wavefront shaping, one could compensate for tissue scattering and increase the imaging penetration depth. When the appropriate illumination wavefront is generated by the SLM, scattered light can be focused in a single spot [1], as illustrated in Figure 1(b). By refocusing the spot at different positions, raster-scan imaging in scattering media could be achieved using wavefront shaping. Finally, combining the wavefront shaping with either acoustic [2], [3] or fluorescence feedback mechanisms [4], [5] enables noninvasive focusing and imaging inside the scattering sample.

Typically, commercially available SLMs rely on liquid crystals or micro electro-mechanical systems (MEMS) [6]. However, substantial developments on the SLM hardware are necessary to unlock *in vivo* imaging applications of wavefront shaping. Improvements should be made on the device modulation rate because of rapidly changing scattering in living tissue, which can be on the order of 1 ms [7], and on system compactness because of the interest of performing experiments in freely moving animals [8]. A recent demonstration by the authors shows that a photonic integrated circuit, more specifically an OPA, can be used for wavefront shaping in scattering media [9]. Additionally, the same work demonstrates that OPAs can outperform conventional spatial light modulators when it comes to the pixel pitch, modulation rate, and device compactness.

In this work, we present a detailed experimental comparison of three different OPA designs. We investigate the influence of array dimensionality (1D vs 2D), array pitch, and antenna emission profile on the wavefront shaping performance. From this, design trade-offs are highlighted. The OPA performance is evaluated for light focusing through tissue-like forward-scattering samples of variable thickness. Intensity enhancement *η* and focusing field of view (FoV) are used as the performance metrics in this study. The working wavelength of the devices is *λ* = 852 nm. This wavelength was chosen because it allows the use of the photonic integrated circuit (PIC) together with fluorescence or photoacoustic feedback mechanisms. Combining the PIC wavefront control and mentioned feedback techniques can allow for noninvasive intratissue focusing and imaging at depth. Finally, imec’s silicon nitride (SiN) platform used for OPA fabrication is suitable for both the NIR and VIS spectral range, both relevant for the proposed feedback techniques.

The article is organized as follows: we first introduce three different OPAs used in the experiments. Afterward, we explain the experimental methods and experimentally compare performance of the OPAs and observe two distinct operation modes. Finally, we discuss the trade-offs in the OPA design and their influence on the performance.

## 2 OPA design

The optical phased arrays consist of an input waveguide, a power splitting section realized as a multimode interferometer tree, a section that performs the phase control, and finally, the antenna array, i.e., the aperture that radiates the light out of the chip, as illustrated in Figure 1. The phase modulators rely on the thermo-optic effect in SiN waveguides, meaning that the phase is modulated by running current through a heating element in the waveguide’s proximity.

**Figure 2:**
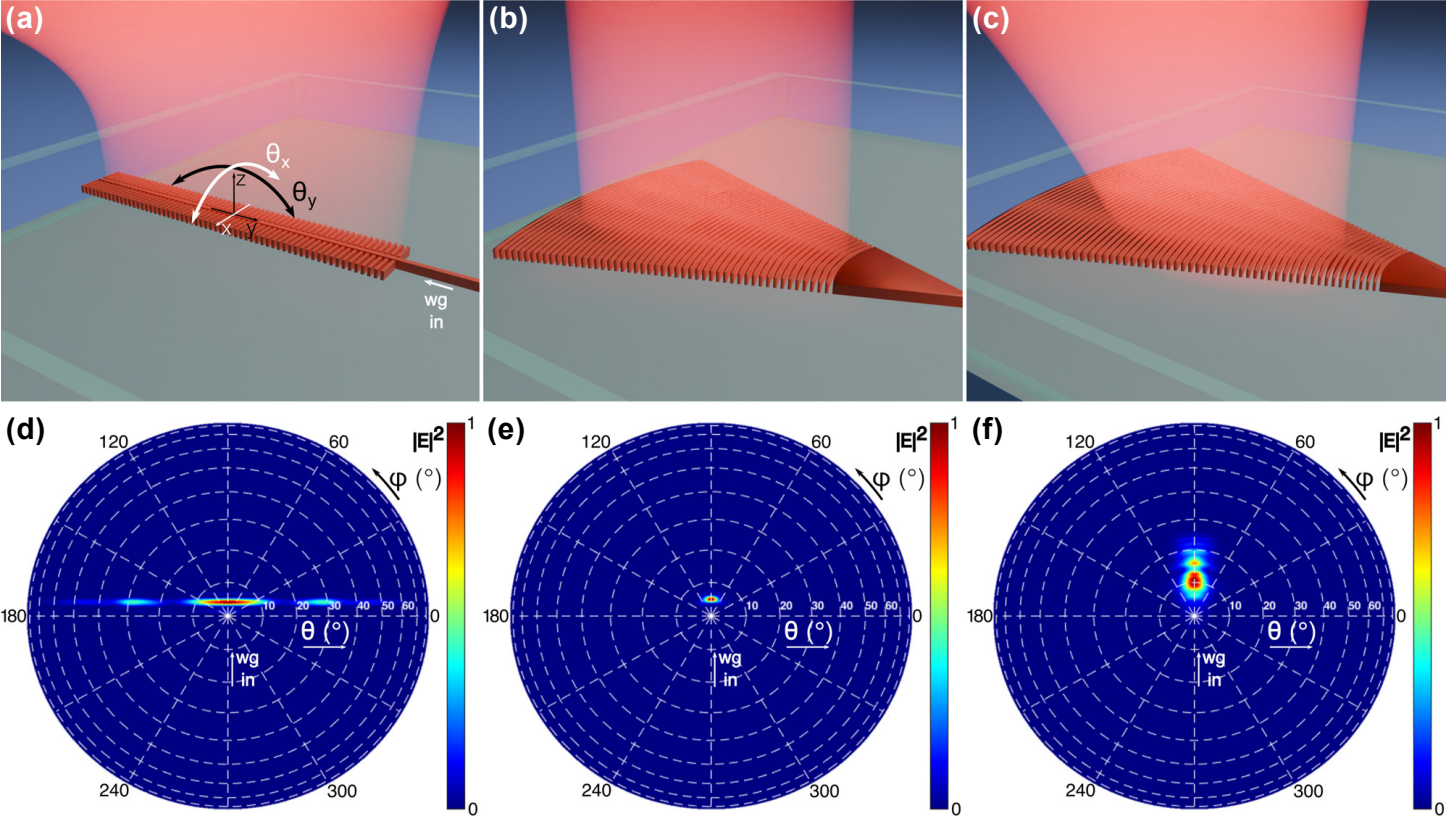
Illustration of the antennas used in the OPAs (a)–(c). (a) Leaky-wave antenna used in 1D OPA with 128 channels. (b) Collimated antenna used in 2D OPA with 128 channels. (c) Divergent chirped-period antenna used in 2D OPA with 64 channels. (d)–(f) Simulations of the far-field emission corresponding to the antenna illustrated above. The “wg in” label in the figures illustrates the direction of the input waveguide in the simulations. *θ* and *φ* are the spherical coordinates. Angles *θ*_*x*_ and *θ*_*y*_ are defined in panel (a) and used in figures presenting experimental images.

When designing the OPA, one has the freedom to choose the antenna emission profile, array pitch, and dimensionality. We explore the OPA design parameter space in order to define the best-fitting design for wavefront shaping applications. The three OPAs presented in this work use antennas with different emission profiles. The one-dimensional OPA uses leaky-wave antenna (LWA) illustrated in Figure 2(a). Since the LWA aperture is elongated over the *y*-axis, it emits a beam collimated in that direction and divergent over the *x*-axis. Except for the main antenna lobe, centered around *θ* ≈ 4°, the far-field radiation has additional side lobes visible in Figure 2(d). The 2D OPA with 128 antennas uses a far-field focusing grating coupler shown in Figure 2(b), which will be called collimated antenna in this work. Its emission is centered around *θ* ≈ 5°, as shown in Figure 2(e). Finally, the 2D OPA with 64 antennas makes use of a chirped-period grating, which emits a more divergent beam compared to the far-field focusing grating. The chirped-period antenna, illustrated in Figure 2(c), emits approximately in the range *θ* = 0° to *θ* = 25°, with the strongest lobe located at *θ* ≈ 10° (Figure 2(f)).

**Figure 3:**
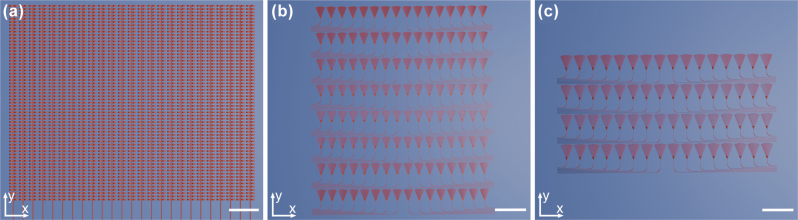
Illustrations of the apertures of the OPAs used for focusing through scattering media. (a) 1D OPA with 128 LWAs placed at the pitch of 5 µm (scale bar length 15 µm). The illustration doesn’t show all 128 antennas present in 1D OPA for detail visibility. (b) 2D OPA with 128 collimated antennas. The values of OPA pitch over *x*-axis and *y*-axis are 35 µm and 84.4 µm, respectively (scale bar length 100 µm). (c) 2D OPA with 64 divergent, chirped-period antennas. The values of OPA pitch over *x*-axis and *y*-axis are 43 µm and 96 µm, respectively (scale bar length 100 µm).

Optical apertures, i.e., regions where the light is radiated out of the OPA using grating emitters, of the fabricated devices are illustrated in Figure 3. In case of the 1D OPA, array pitch is 5 µm, and the antennas are 1.1 mm long. Such length of the LWA was chosen to ensure that almost all of the light is radiated out of the waveguide ( ≥99 % according to the simulations). In case of 2D OPAs, the array pitch must increase to accommodate for more complex waveguide routing [10]. To minimize the array pitch in 2D OPAs, an advanced low-loss waveguide bend with variable waveguide width was used [11].

In the 2D OPA with 128 elements, collimated antennas were placed at a *y*-axis pitch of 84.4 µm and *x*-axis pitch of 35 µm (coordinate system illustrated in Figure 3). Similar pitch values, namely 96 µm over *y*-axis and 43 µm over *x*-axis, characterize the 2D OPA with 64 elements. We note that the 2D OPA with chirped-period antennas contains less elements because of limited design area available. Nevertheless, even with a smaller-scale OPA, relevant experimental conclusions can be drawn. Simulated upwards radiation efficiency is 56 % and 53 % for the collimated antenna and chirped-period antenna, respectively. For the LWA, simulated upwards radiation efficiency is 36 %. All the antenna simulations were performed using Ansys Lumerical FDTD. Finally, all the OPAs use phase shifters based on the thermo-optic effect with measured modulation efficiency of 20 mW/π, and all the devices were fabricated in imec’s 200 mm SiN PIC platform [12] (BioPIX300).

## 3 Experimental methods

**Figure 4:**
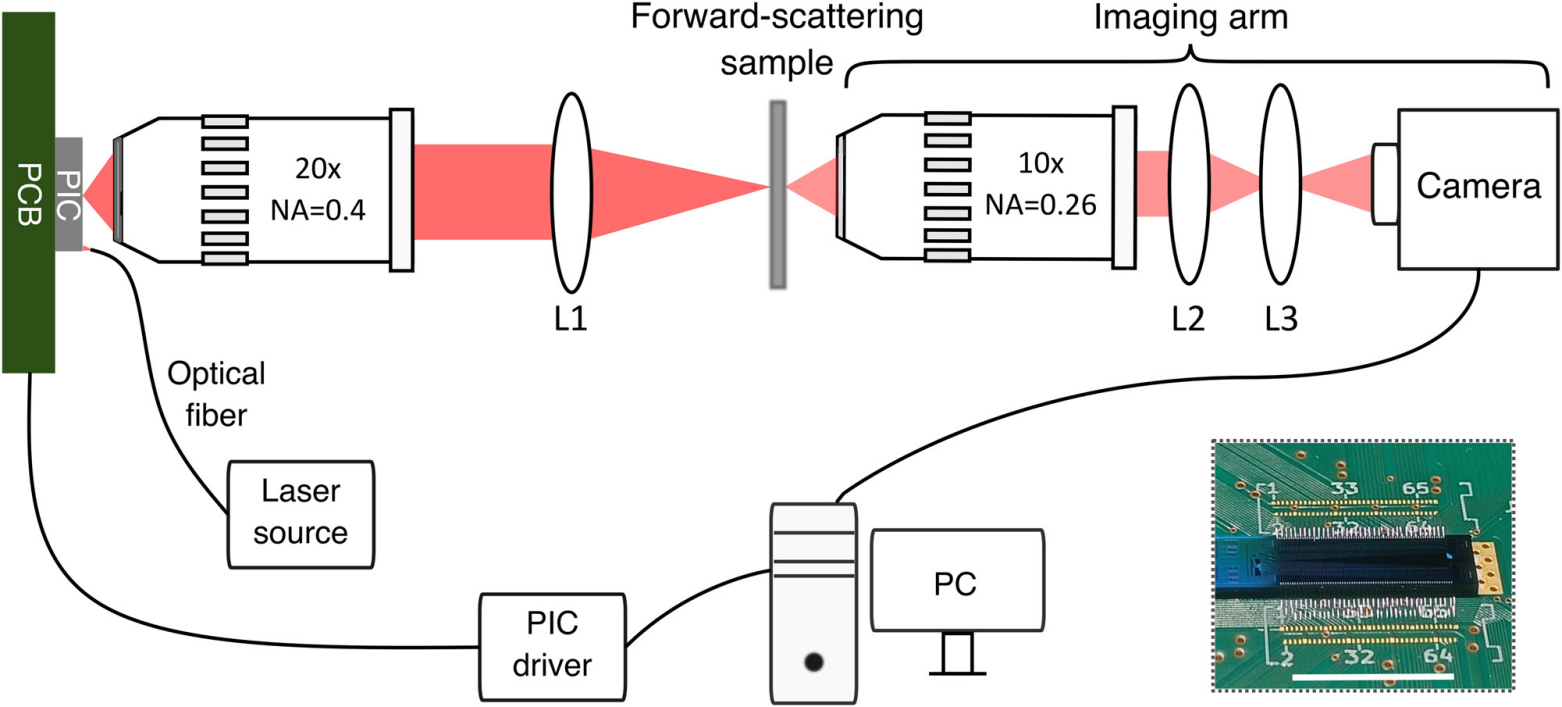
Experimental setup used for focusing through forward-scattering samples. Light emitted from the photonic integrated circuit (PIC) is imaged on the scattering sample by means of a 20× objective with the focal length of *f*_obj_ = 10 mm, and lens L1 (*f*_*L*1_ = 25 mm). Far field of the light scattered by the sample is imaged using a 10× objective, Bertrand lens (L2, *f*_*L*2_ = 60 mm) and a tube lens (L3, *f*_*L*3_ = 100 mm) onto the camera. The readout value of a single camera pixel is the feedback signal for the PIC phase-optimization algorithm running on a PC. The PIC is wire bonded to a printed circuit board (PCB), which provides the electrical connection to the PIC driving electronics. Inset shows an image of a wavefront shaping PIC wire bonded on a PCB (scale bar length is 1 cm).

Experiments were performed using the setup shown in Figure 4. Light from an external laser source (Thorlabs DBR852PN) is coupled into the PIC, which is wire bonded on a PCB. The purpose of the PCB is establishing electrical connections between the PIC and the PIC driver used for controlling the phase modulators. OPA’s aperture was imaged onto the forward-scattering sample, consisting of a variable number of layers of Parafilm M. Such a sample was chosen because it has scattering properties [5] similar to biological tissue [13], [14]. The far-field of scattered light was imaged onto the camera (Allied Vision Goldeye G-130) using an objective, Bertrand lens, and tube lens. The Bertrand lens is inserted between the objective and tube lens to enable projection of the objective’s back focal plane, which corresponds to the far-field intensity distribution, on the camera’s sensor. The far-field image also represents the angular distribution of the light scattered by the sample. Thus, by imaging the far field we have insight into the angular cone in which the scattered light propagates after the sample. By increasing the sample thickness, i.e., increasing the number of Parafilm M layers, we investigate the extent of the focusing (angular) FoV at different depths in a forward-scattering medium.

Additionally, we also observe two distinct wavefront shaping regimes dependent on the sample thickness. Scattering properties of Parafilm M were previously characterized [5], and it was found that the scattering mean free path is *l*_*s*_ ≈ 170 µm and that the transport mean free path, i.e., distance after which light transport enters the diffusion regime, is *l*_*t*_ ≈ 720 µm. For reference, the thickness of a single Parafilm M layer is approximately 120 µm. Enabling high-resolution imaging at a depth greater than *l*_*t*_ in tissue, or approx. six Parafilm M layers in our experiments, is especially interesting for wavefront shaping, as the conventional microscopy techniques, such as two-photon or confocal microscopy, fail to form an image at those depths [15]. Stepwise sequential iterative optimization is employed to determine the optimal OPA wavefront. Readout value of a single camera pixel is used as the feedback signal during the optimization. A detailed description of the optimization algorithm can be found in [16].

Please note that the optimized focusing wavefront is valid only inside a bandwidth [17], determined by the scattering medium’s thickness and scattering properties [18]. However, for our target application – fluorescence feedback-based focusing for imaging, a narrow illumination bandwidth (laser line) is sufficient [5], [19].

## 4 Results and discussion

Using the setup explained in the previous section, we compare OPA performance when focusing through scattering samples of different thicknesses.

### 4.1 Focusing field of view

**Figure 5:**
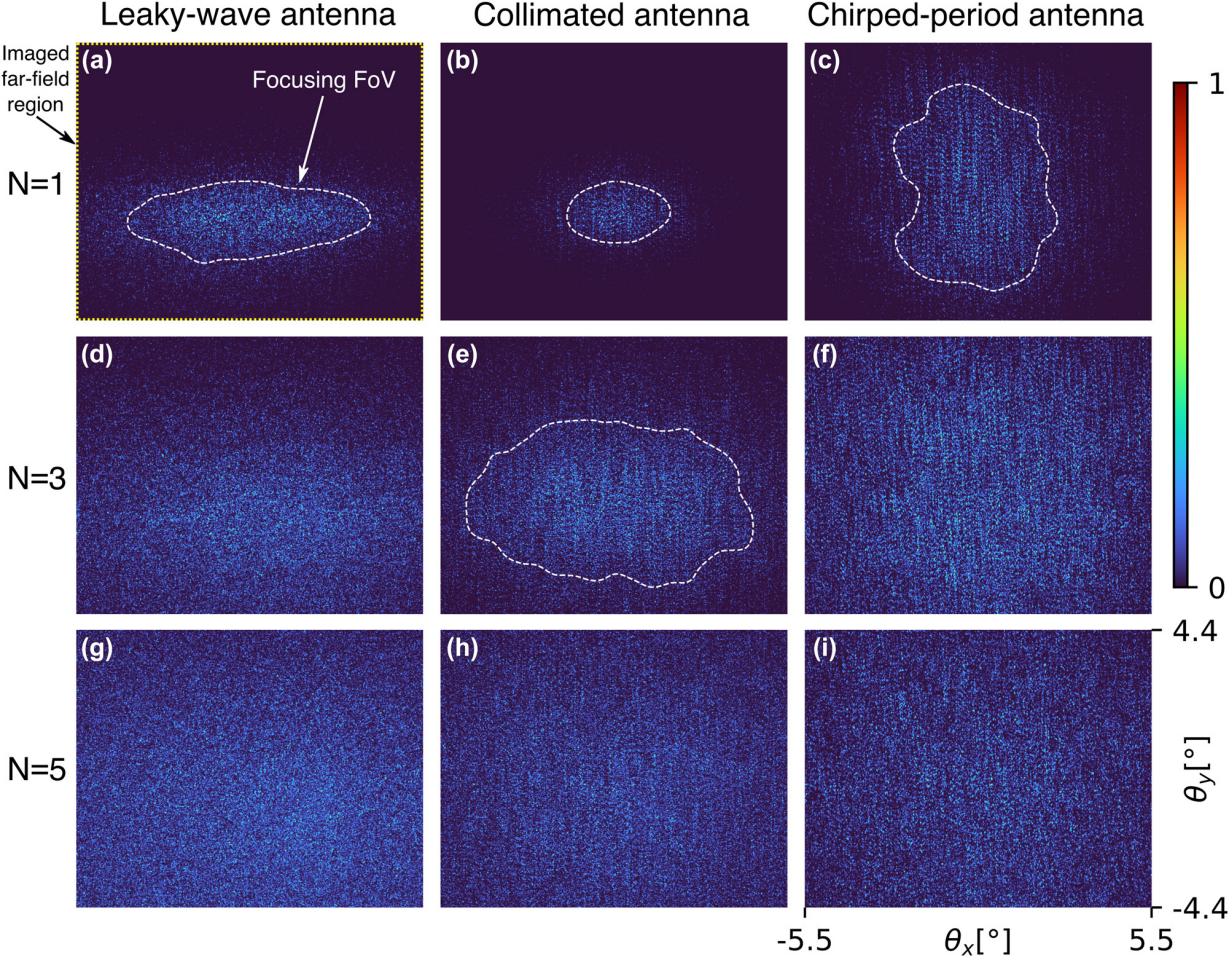
Experimental data on the angular FoV in which efficiently focusing the scattered light is possible, i.e., focusing FoV (indicated by the white line in panels (a–c), (e)). Evolution of the focusing FoV is shown for different number of Parafilm M layers (*N*) used as the scattering sample and different OPA antenna types (illustrated in Figure 2). The chirped-period antenna illuminates the whole imaged far-field region (indicated by the yellow line in panel (a)) in a thinner sample (*N* = 3), while the collimated antenna and LWA only fill the whole imaged far-field region in the thicker sample (*N* = 5). Angles *θ*_*x*_ and *θ*_*y*_ are the inclination angles measured along *x* and *y* axis, respectively (illustrated in Figure 2(a)). The color bar refers to the light intensity in all the images in this figure. For the chirped-period antenna, the camera sensor position was vertically adjusted to ensure that all the main lobes are in the imaged region.

The first observation is related to the size of the focusing FoV (angular region indicated by the white line in Figure 5(a)), being the strongly illuminated part of the imaged far-field region before applying any wavefront control. The term focusing FoV was chosen because efficiently focusing the scattered light, and raster-scan imaging, will be possible only in the region where the speckle illumination intensity is non-negligible. Therefore, the focusing FoV shown in Figure 5 is equivalent to the achievable imaging FoV using wavefront shaping. We experimentally investigate the evolution of focusing FoV for different sample thicknesses, representing different depths in the biological tissue.

Figure 5 shows the angular extent of the initial speckle for different sample thicknesses and different OPAs. As expected, with increasing sample thickness, i.e., number of Parafilm M layers *N*, photons experience more scattering events, and the focusing FoV consequently increases. However, the antenna emission profile has an influence on the extent of the focusing FoV as well. To quantitatively confirm this for the sample with *N* = 1 Parafilm M layer, we define the focusing FoV by the contour where the moving-average speckle intensity gets reduced by the factor 1/*e* compared to its maximum. The contour is indicated with a dashed white line in Figure 5(a)–(c), (e). For the leaky-wave antenna (panel (a)), we measure FoV extent of Δ*θ*_*x*_ = 7.7° and Δ*θ*_*y*_ = 2.5°, over *θ*_*x*_ and *θ*_*y*_-axis, respectively. For the collimated (panel (b)) and chirped-period (panel (c)) antenna, we measure FoV extents of (Δ*θ*_*x*_, Δ*θ*_*y*_) = (3.3°, 1.9°) and (Δ*θ*_*x*_, Δ*θ*_*y*_) = (5.2°, 6.5°), respectively. We observe that for the same sample thickness, the OPA with chirped-period antennas has the highest Δ*θ*_*x*_∗Δ*θ*_*y*_ product, i.e., the widest focusing FoV, while the collimated antenna has the smallest focusing FoV. Thus, a divergent antenna can illuminate the whole imaged far-field region (indicated by the yellow line in Figure 5(a)) in thinner samples. This can be confirmed in Figure 5(f) where the chirped-period antenna illuminates the sample with *N* = 3 layers of Parafilm M. Differently, the collimated antenna and LWA fill the full imaged far-field region only in thicker samples, e.g., *N* = 5 (Figure 5(h)).

To quantitatively confirm that the focusing FoV increases with increasing sample thickness, we compare its (Δ*θ*_*x*_, Δ*θ*_*y*_) extents for the collimated antenna at different sample thicknesses. For this antenna, we measure (Δ*θ*_*x*_, Δ*θ*_*y*_) = (3.3°, 1.9°) and (Δ*θ*_*x*_, Δ*θ*_*y*_) = (9.1°, 5.3°), for *N* = 1 (Figure 5(b)) and *N* = 3 (Figure 5(e)), respectively. We can conclude that the focusing FoV increases with increasing sample thickness and with increasing OPA antenna divergence. Therefore, when designing the OPA for maximal focusing FoV, a more divergent antenna would be preferred as it offers possibility to perform the wavefront shaping in a wider focusing FoV. However, if one designs the OPA for maximal light outcoupling efficiency, a more collimated antenna is preferred because it has higher simulated upwards radiation efficiency, as discussed in the OPA Design section.

### 4.2 Two focusing regimes

**Figure 6:**
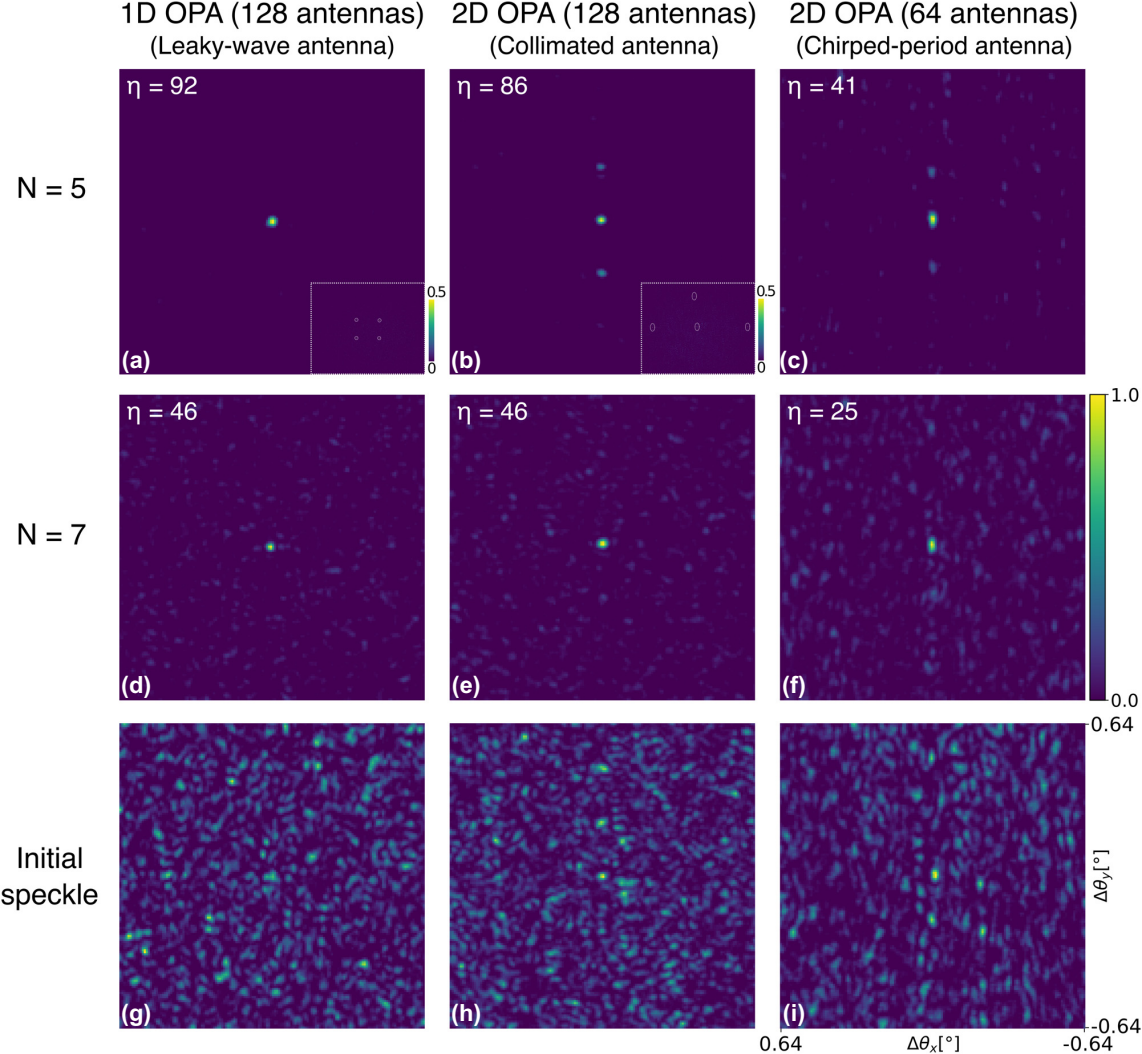
Overview of two regimes when focusing through forward scattering media. (a–c) First regime: images of optimized focal spots for all OPAs when the scattering sample is thinner than the transport mean free path *l*_*t*_ (*N* = 5). Additional grating lobes are present in experiments with 2D OPA because of residual ballistic photons. Obtained enhancement is shown in the panel’s top left corner. (d–f) Second regime: images of optimized focal spots when the scattering sample is thicker than *l*_*t*_ (*N* = 7). No additional grating lobes are present. (g–i) Initial speckle before the wavefront optimization for *N* = 5. Color bar corresponds to images in each panel, while each image is normalized with respect to its maximum value. Angles *θ*_*x*_ and *θ*_*y*_ are the inclination angles measured along *x* and *y* axis, respectively (illustrated in Figure 2(a)). Relative angles (Δ*θ*_*x*/*y*_) with respect to the center of the optimized focus are shown. Single pixel in all the images corresponds to the *θ* increment of 0.0086°. Insets in the bottom right of (a) and (b) show beam raster scanning. Dashed white lines highlight different focus positions in the raster scan. Insets are generated by summing the individual images of different focused spots. Insets’ angular *θ*_*x*/*y*_ span is the same as in Figure 5, and they have dedicated scale bars for better spot visibility.

Images of focusing through forward-scattering samples of different thicknesses using the three previously described OPAs are shown in Figure 6. The initial speckle pattern before wavefront optimization is shown in the same figure (panels (g), (h), (i)) for the sample with *N* = 5. The enhancement of the focused spot *η* is defined as the ratio of the optimized focus intensity (*I*_focus_) and the average intensity in the initial speckle pattern (*I*_init_): *η* = *I*_focus_/*I*_init_. The achieved *η* values are shown in Figure 6 for different sample-OPA combinations. For a phase-modulated wavefront, it was shown theoretically that the maximum achievable enhancement [20] is *η*_max_ = *π*/4∗(*N*_*s*_ − 1) + 1, where *N*_*s*_ is the number of wavefront segments, i.e., the number of antennas in the OPA. For our sample with five Parafilm M layers (*N* = 5), the experimentally achieved enhancement for the 1D OPA with 128 antennas is *η* = 92, which is close to the theoretical maximum *η*_max_ ≈ 101 for the given number of antennas. A similar focus enhancement of *η* = 86 is achieved using the 2D OPA with 128 antennas in the same scattering sample. Finally, the 2D OPA with 64 chirped-period antennas achieved a lower enhancement value of *η* = 41 because of the lower number of antennas used for wavefront control. Consequently, more background speckle is visible around the optimized focus in this case. Nevertheless, the achieved *η* value is close to its theoretical maximum *η*_max_ ≈ 50 for using 64 antennas.

The optimized images for the two 2D OPAs and *N* = 5 contain two pronounced regularly spaced spots above and below the main focus (Figure 6(b) and (c)), while the intensity in the central focal spot was the only feedback signal during wavefront optimization. As we will show, these extra spots are a consequence of residual ballistic photons in the sample, in combination with a bigger 2D array pitch.

As the scattering sample (*N* = 5) is thicker than the scattering mean free path (*l*_*s*_), but thinner than the transport mean free path (*l*_*t*_), light propagation isn’t yet in the diffusive regime and the number of ballistic photons is non-negligible (*l*_*s*_ and *l*_*t*_ values given in Section 3). To confirm that the extra spots are, in fact, OPA grating lobes, which arise because of the ballistic photons, we will use the theory of OPAs, which considers propagation in vacuum, i.e., ballistic photons only, discussed in chapter 3.1.5 of [21].

A known result from the theory of phased arrays is that the angular spacing of the array grating lobes in case of array pitch *δ* > *λ*/2 is Δ*θ*_OPA_ = arcsin(*λ*/*Mδ*), where *λ* is the wavelength and *M* is the magnification of the arm, which images the OPA on the scattering sample. The magnification *M*, included in the formula above to account for our experimental setting in which the OPA is imaged onto the scattering sample, can be calculated using *M* = *f*_*L*1_/*f*_obj_ = 2.5, where *f*_*L*1_ = 25 mm and *f*_obj_ = 10 mm are the focal lengths of the lens L1 and the objective, respectively. By applying the formula above to the 2D OPA with 64 antennas placed at the *y*-axis pitch of *δ*_*y*_ = 96 µm, we get that the *y*-axis grating lobe spacing equals Δ*θ*_OPA*y*_ = 0.203°. The angular spacing of the extra spots in Figure 6(c), corresponding to the experiment using the 2D OPA with 64 antennas and *N* = 5, is Δ*θ*_*y*_ = 0.206°. Thus, the spacing of experimentally observed extra spots Δ*θ*_*y*_ and the calculated OPA grating lobe spacing Δ*θ*_OPA*y*_ are in good accordance. This result confirms that the extra spots in Figure 6 are OPA grating lobes formed by the ballistic photons. Similar result is obtained when considering the 2D OPA with 128 channels.

In addition, we characterize the power of OPA grating lobes observed in Figure 6(b) and (c) using the side lobe suppression ratio (SLSR), defined as SLSR = 10 log_10_(*I*_focus_/*I*_lobe_), where *I*_lobe_ is the peak intensity of the strongest grating lobe. SLSRs of 3.1 dB and 4.6 dB are measured for 2D OPAs with 128 and 64 antennas, respectively. We expect that the grating lobes visible in Figure 6(b) and (c) can be suppressed by using nonuniform OPA pitch (sparse arrays e.g.) [10], [22]. These architectures mitigate periodicity-induced grating lobes in free space beam steering and would have the same effect in wavefront shaping because we observe grating lobes formed by ballistic (nonscattered) photons. Additionally, sparse arrays allow for denser antenna placing in 2D OPAs, requiring less chip area.

As the angular spacing of the OPA grating lobes is reciprocally related to the OPA pitch, by reducing the pitch, the angular spacing of grating lobes is increased. Light rays, which propagate through a sample of thickness *t* at different angles Δ*θ*, acquire a relative phase difference Δ*φ*. The difference in phase Δ*φ* increases with increasing Δ*θ* and *t*. With a big enough Δ*φ*, the wavefront correction valid for the main lobe (optimized spot) isn’t valid for the OPA grating lobe. Therefore, grating lobes propagating at higher Δ*θ* get suppressed in our experiments. Accordingly, the 1D OPA with significantly smaller pitch doesn’t exhibit extra spots, i.e., grating lobes, when focusing. Thus, by reducing the OPA pitch, grating lobes surrounding the optimized focus can be suppressed, as they drift out of phase with respect to the central focus. The observed effect can also be related to the memory effect in scattering media, where the correlation FoV inversely scales with the scattering sample’s thickness [23], [24].

The insets in Figure 6 (top row) illustrate the ability to do beam steering, or raster scanning, in the case of 1D and 2D OPA with 128 antennas and *N* = 5. This also allows us to characterize the *η* over the FoV. The average enhancement for the 1D OPA is *η* = 70 ± 4. The 2D OPA achieves average *η* over the FoV of *η* = 67 ± 3. Therefore, 1D and 2D OPA with the same antenna count achieve very close, almost identical *η* values over the FoV. Finally, a roll-off in the *η* values is expected at the very edge of the focusing FoV, because there are fewer photons available for focusing at this location. This is similar to the free-space OPA theory, where the far-field beam intensity reduces at the edges of the steering range because of a roll-off in the antenna emission strength (see chapter 3 of [21]).

When focusing through seven layers of Parafilm M (Figure 6(d)–(f)), the achieved enhancements are lower than in the previous sample. Therefore, background speckle is more visible. The enhancement reduction could be attributed to a shorter speckle persistence time in the thicker sample. Speckle persistence time is the time scale over which the speckle pattern remains stable, and it is influenced by changes in the sample. These changes could be induced by, e.g., temperature drifts that vary the sample’s scattering characteristics. It was previously demonstrated that reducing the speckle persistence time results in enhancement reduction [1]. As the sample thickness is greater than *l*_*t*_ (*N* = 7), light propagation is in the diffusive regime. We observe that the grating lobes, which were present when characterizing 2D OPAs for *N* = 5, are suppressed in the thicker sample. We can observe that both the 1D OPA and 2D OPA with 128 antennas reach enhancement values of *η* = 46. As expected, the 2D OPA with only 64 antennas reaches a lower enhancement value of *η* = 25.

The focus size in Figure 6 is diffraction limited and defined by the size of the OPA aperture as previously shown in [9], or more generally speaking by the extent of the illumination impinging on the scattering sample [25]. Consequently, the OPA aperture aspect ratio defines the focal spot aspect ratio in our experiments. In case of 1D and 2D OPAs with 128 antennas, the OPA aperture aspect ratio is close to unity, resulting in a round focal spot (Figure 6(a), (b), (d) and (e)). We measure the spot full width at half maximum (FWHM) over *θ*_*x*_ and *θ*_*y*_ axis for these two OPAs. For *N* = 5, the 1D OPA generates a round spot characterized by ${FWHM}_{\theta_{x}}={FWHM}_{\theta_{y}}=$ 0.034°. In the same scattering sample, we measure ${FWHM}_{\theta_{x}}={FWHM}_{\theta_{y}}=$ 0.026° for the 2D OPA with 128 antennas. The same trend is followed by these two OPAs for *N* = 7. Differently, the focus is elongated over *θ*_*y*_ in case of the 2D OPA with 64 antennas (Figure 6(c) and (f)). For this OPA, we measure the ratio of spot FWHM over *θ*_*y*_ and *θ*_*x*_ axis of: $\frac{{FWHM}_{\theta_{y}}}{{FWHM}_{\theta_{x}}}\approx 2$ . Measured spot aspect ratio is inversely proportional to the OPA’s aperture aspect ratio of *a*_*y*_/*a*_*x*_ ≈ 0.5 (Figure 3(c)), where *a*_*x*_, *a*_*y*_ are the aperture sizes over *x* and *y*-axis, respectively. This observation is also in agreement with the diffraction theory [25], as a narrower aperture results in a wider far-field spot.

## 5 Conclusions

In the presented work, we experimentally explored the design parameter space of OPA for focusing the light scattered by tissue-like samples and observed two distinct operation regimes. In the first regime, the light is scattered by a sample whose thickness *t* is *l*_*s*_ < *t* < *l*_*t*_ – meaning that the sample is not thick enough to reach the diffusive light propagation mode. In this regime, we experimentally observe a clear difference between focusing using a 1D or a 2D OPA. Because of an increased array pitch in the 2D OPA case and a non-negligible number of ballistic photons, we observe additional regularly spaced grating lobes surrounding the optimized focus. These grating lobes form a multispot focusing pattern, which could still be used for raster scan imaging at depth. However, this will impose more load to the image reconstruction algorithm, as it needs to account for a more complex illumination pattern. Differently, experiments using a 1D OPA with significantly smaller pitch show a single focus with no grating lobes. Suppression of grating lobes at higher angles can be related to the memory effect in scattering media [23]. Regarding the intensity enhancement, 1D and 2D OPA with the same number of antennas achieve comparable *η* values in this scattering regime.

When focusing in the second, diffusive regime, no surrounding grating lobes are observed next to the focus, neither for 2D nor 1D OPA. We observe that, also in this case, the achieved enhancement depends only on the number of antennas in the OPA, and not on the array dimensionality (1D or 2D). The focus size is diffraction limited and determined by the extent of the beam that illuminates the scattering sample [25], which is defined by the size of the OPA aperture in our experiments [9]. In order to achieve the same imaging resolution over *x* and *y*-axis, a symmetric OPA aperture is preferred. We note that, thanks to the 1D-to-2D transformation performed by the scattering medium [26], a 1D OPA can be used for focusing the scattered light over a 2D grid, as previously demonstrated [9]. This enables a 2D raster scan of the focal spot with a 1D OPA. Given the experimental results, a 1D OPA is the preferred choice because of simpler waveguide routing, smaller array pitch, and single-spot focusing in both regimes. We note that, differently from a 1D array, the array pitch in 2D OPA scales with the array size to accommodate for more complex waveguide routing [10].

Finally, we demonstrated that the OPAs that employ antennas with divergent emission maximize the imaging FoV, while the OPAs that employ collimated antennas maximize the light outcoupling efficiency. We note that the antenna emission efficiency has an influence on the total PIC insertion losses, but not on the achievable enhancement *η*. In scattering media, *η* depends on *N*_*s*_ (OPA antenna count), as observed in our experiments and as predicted by the random matrix theory [20]: *η* = *π*/4∗(*N*_*s*_ − 1) + 1. Therefore, for the applications with a limited photon budget, total PIC insertion losses can be reduced by using a more efficient, collimated antenna. However, this comes with a FoV reduction trade-off. In future, increasing the number of antennas in the OPA, no matter the FoV size and OPA dimensionality (1D vs 2D), will allow for higher experimental *η* [9], [20].
